# Transcatheter Arterial Chemoembolization Imaging Features in MR-Linac Radiation Therapy Planning for the Liver

**DOI:** 10.7759/cureus.50459

**Published:** 2023-12-13

**Authors:** Jennie Crosby, Michael F Bassetti, Newton J Hurst, Tera Kruser, Carri K Glide-Hurst

**Affiliations:** 1 Department of Human Oncology, University of Wisconsin - Madison, Madison, USA; 2 Department of Radiation Oncology, University of Wisconsin Hospitals and Clinics, Madison, USA

**Keywords:** radiation therapy treatment planning, adaptive radiation therapy, liver sbrt, mr-guided radiation therapy, mr-linac, transcatheter arterial chemoembolization (tace)

## Abstract

For MR-guided radiation therapy treatment planning, an MRI and CT of the intended treatment site are typically acquired. Patients’ prior treatments or procedures can cause image artifacts in one or both scans, which may impact treatment planning or the radiation dose calculation. In this case report, a patient with several previous transcatheter arterial chemoembolization (TACE) procedures was planned for radiation therapy on a low-field MR-linac, and the impact of residual iodinated oil on the radiation dose calculation and MR-guided adaptive workflow was evaluated.

## Introduction

MR-guided adaptive radiation therapy (MRgART) can be helpful in the treatment of primary and secondary liver tumors, allowing for tumor dose escalation while enabling decreases in dose to critical surrounding organs at risk (OARs), reducing the potential for toxicity [1-3]. In addition, target margin reduction is possible through gated breath hold treatment delivery [4-6] that minimizes diaphragm-induced liver motion [7], shown to be on the order of 1 cm for shallow breathing [8-11] and up to 5 cm for deep breathing [10,11].

Online MRgART and respiratory gating performed on internal anatomy at eight frames per second in the sagittal plane can be achieved by the 0.35T ViewRay MRIdian MR-linac system (ViewRay Inc., Oakwood, USA). For the MRgART treatment planning workflow, during the initial simulation process, an MR image of the patient is acquired with the 0.35T scanner, and then a CT image is acquired on a CT simulator for co-registration to obtain electron density information needed for radiation dose calculation. Following the acquisition of these images, the radiation oncologist contours the target(s) and nearby OARs. After contouring is performed, a dosimetrist plans the radiation treatment that is then approved by the radiation oncologist, after which a treatment plan review is performed by a medical physicist. When the patient returns for each treatment fraction, a new MR scan is acquired, and the electron density information from the original CT image is deformed to the new MR to enable daily dose calculation. The OARs near the target are contoured, and the dose is recalculated based on the new position of the patient’s anatomy. If clinically indicated, there is the opportunity to reoptimize the dose to improve target coverage and/or reduce doses to OARs to achieve clinical planning goals.

In our MRgART practice, often patients undergoing this treatment have received prior treatments, such as prior radiation, chemotherapy, and/or ablation, among others.

## Case presentation

For this case, an 80-year-old male patient with multifocal hepatocellular carcinoma (HCC) had a predominant larger mass involving the right posterior liver segments VI and VII and a smaller mass in liver segment 4A. He was staged as Stage IIIA (T3 N0 M0) HCC, with a Childs-Pugh score of A5, and was planned to be treated with stereotactic body radiation therapy (SBRT) with MRgART to both liver masses. The patient underwent treatment planning scans first receiving the gadolinium-based contrast agent gadoxetate (EOVIST, Bayer HealthCare Pharmaceuticals, Berlin, Germany) and then proceeding with scanning on the 0.35T ViewRay MRIdian (ViewRay Inc., Oakwood, USA) and on a Siemens SOMATOM Edge CT (Siemens Healthcare Inc., Erlangen, Germany). At the time of these planning scans, the patient had already undergone multiple prior treatments, including three transcatheter arterial chemoembolization (TACE) procedures and a hepatic radioembolization with Y-90.

**Figure 1:**
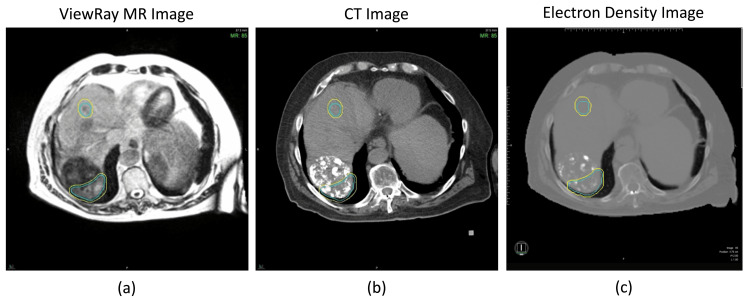
MRI image acquired on 0.35 T ViewRay MRIdian (a) and CT image acquired on Siemens SOMATOM Edge (b) for use in radiation therapy treatment planning of two liver lesions. The corresponding electron density (c) is used for the radiation dose calculation. The blue volumes indicate the gross tumor volumes (GTVs), while the yellow indicates a 3 mm expansion to form the planning target volume (PTV). Note the appearance of a highly attenuating (bright) material in the CT image.

MR images were acquired for treatment planning with no significant imaging artifacts present, although heterogeneity within the larger target was noted. However, upon acquisition of the treatment planning CT, bright artifacts indicative of a highly attenuating material were observed within the region of the intended treatment (Figure 1(b)).

Lim et al. published a pictorial essay about imaging features of HCC after TACE [12], which assisted in the identification of the bright material in the CT images. Iodinated oil is used in the TACE procedure, mixed with anticancer agents, and the accumulation pattern of the iodinated oil on CT is used to assess the effects of TACE [12]. MR signal intensity is not influenced by the presence of the iodinated oil from TACE; however, due to the presence of iodine, the iodinated oil was greatly enhanced on the CT [12]. As a result, the CT used for electron density information represented the iodinated oil as a highly attenuating material.

## Discussion

Given this high attenuation CT artifact and to ensure accurate treatment planning, the iodinated oil on the CT was contoured using a thresholding technique; the average HU value was 307 HU with a standard deviation of 335 HU, a minimum of -317 HU, and a maximum of 3,037 HU. The CT to density table for our Siemens SOMATOM Edge CT gave a density of 1.3 g/cc for approximately 300 HU. Lipiodol was the iodinated oil injected, according to the TACE procedures’ records, which has a density of 1.29 g/cc. Therefore, the average CT reported density was accurate. However, due to the wide range of minimum to maximum HU values, a density override of 1.29 g/cc was applied to the contoured iodinated oil in the treatment planning system. Treatment planning consisted of 17 beams entering through the patient’s anterior, posterior, and right side, to a total dose of 45 Gy in five fractions.

**Figure 2:**
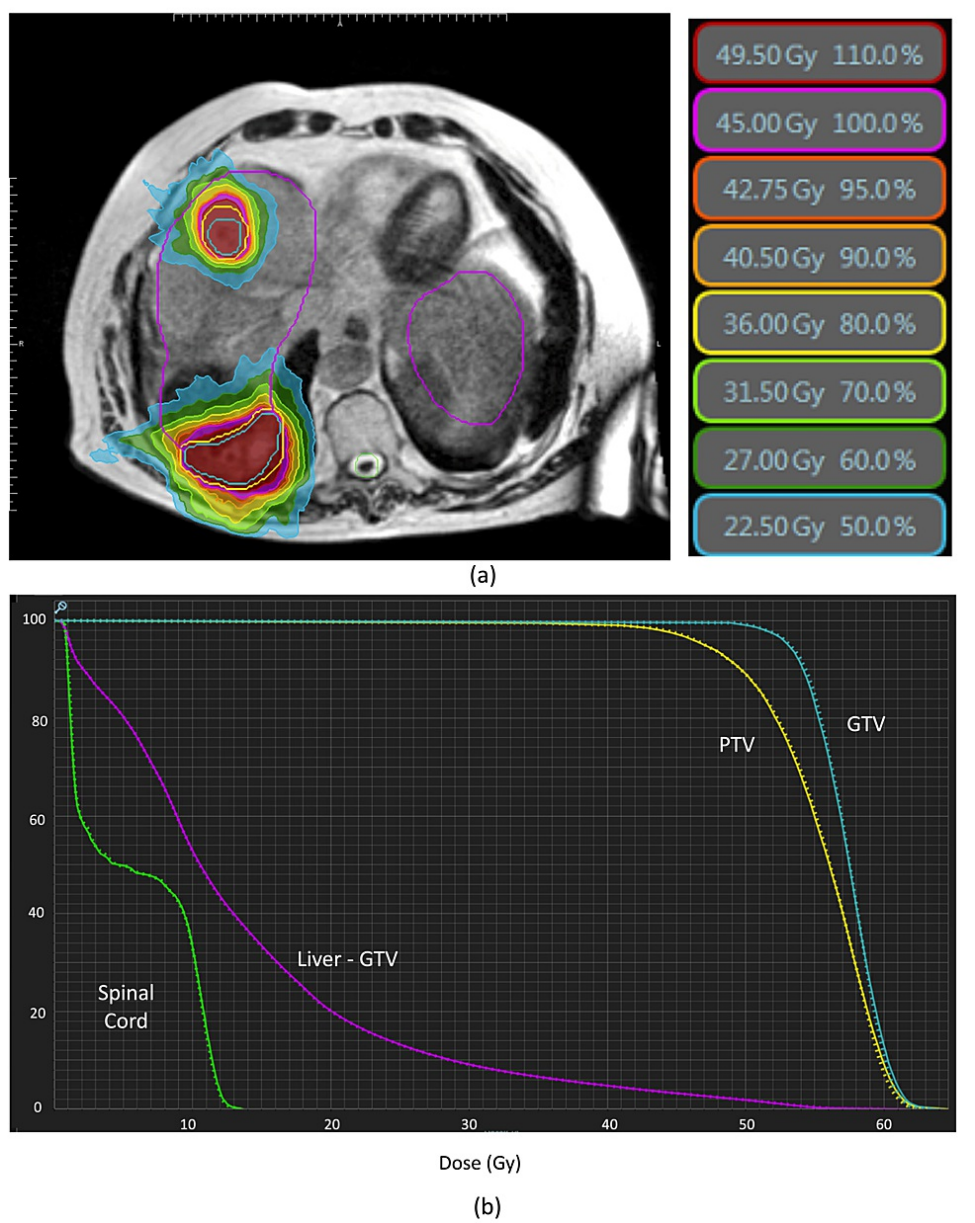
Radiation dose distribution without the density override applied (a) and the dose volume histogram for the targets (GTV and PTV), as well as for the spinal cord and liver-GTV (b). The dashed line is for the plan with the density override applied, and the solid line represents the plan without the density override applied.

To quantify the dosimetric impact of the iodinated oil, the treatment plan was calculated with and without the density override. The coverage of the PTV at the prescription dose (45 Gy) increased by 0.2% for the plan with the override. The coverage of the GTV at 120% prescription dose increased by approximately 1% for the plan with the override. The quantity of Liver-GTV at 15 Gy decreased by 3 cc for the plan with the override (Figure 2). Kawahara et al. [13] performed a similar Monte Carlo dose calculation with and without a lipiodol density override for a patient with prior TACE. They found a maximum point difference of the dose within the PTV of 6%; in our case, we found a maximum point difference of 1%. The quantity of lipiodol within that patient and the beam arrangement are different, which may account for the differences in our findings. However, both results demonstrate a relatively small impact on the dose calculation.

Since the iodinated oil override did not cause a clinically meaningful difference in doses to targets and organs at risk, the plan without the override was selected to use for treatment. In addition, since only an MR image is acquired for each treatment fraction and the original CT is deformed to it, the exact position of the iodinated oil would present workflow and resource challenges to ensure that the density override is applied in the correct location for each treatment fraction.

**Figure 3:**
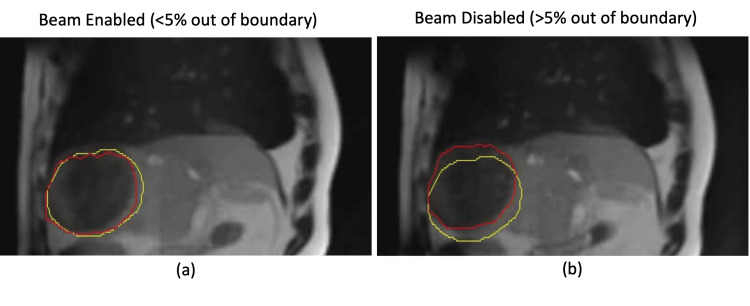
The region being tracked (red) and the boundary (yellow), which is a 3 mm expansion of the tracking structure. When the tracking structure is less than 5% outside the boundary (a), the beam is enabled; however, when the tracking structure exceeds 5% outside the boundary (b), the beam is disabled to prevent radiation delivery when the target is not in the correct position. The presence of iodinated oil in this region did not noticeably impact the tracking performance.

The region containing the iodinated oil was used for tracking and gating the treatment delivery for all five fractions. Figure 3 shows an example of when the region was within the 3 mm boundary and when it was not. The presence of the oil did not seem to appreciably impact the tracking of the region.

## Conclusions

The appearance of residual iodinated oil following prior TACE procedures on CT causes concern about the accuracy of the dose calculation during radiation therapy treatment planning. We found that the presence of the oil was not clinically impactful for a lesion abutting the region by calculating the radiation dose with and without density overrides of the iodinated oil. Due to the limited signal change in the daily MR image, it would have been challenging to precisely account for the position of the oil in each fraction, and thus no additional density overrides were conducted. In addition to having a non-clinically impactful dosimetric impact, the region containing the oil was successfully used for respiratory gating of treatment delivery.
